# Editorial: Biotechnology solutions for crop protection

**DOI:** 10.3389/fpls.2023.1294207

**Published:** 2023-10-26

**Authors:** Jeremy Sweet, Fabio Veronesi

**Affiliations:** ^1^ Sweet Environmental Consultants, Cambridge, United Kingdom; ^2^ University of Perugia, Borgo XX Giugno, Perugia, Italy

**Keywords:** resistance to biotic stresses, new genetic biotechnologies, effects of transgenic crops, weed flora suppression, advanced tools for plant breeding

Resistance or susceptibility to biotic stresses depends on both the genes of the plant and those of the pathogen. Schematically, two situations can be distinguished, one in which the resistance is controlled by one or a few genes and therefore presents itself as a qualitative trait with two phenotypes, susceptible or resistant, and one in which the resistance of the plant is controlled by many genes and presents itself as a quantitative trait. The term “vertical” resistance was introduced for the first situation and “horizontal” resistance for the second, with reference to the graphs representing the level of resistance of different cultivated varieties to different pathotypes or races of the pathogen.

In classical breeding, selection that exploits vertical resistance is essentially based on backcrossing to introduce into susceptible cultivated varieties the specific resistance gene or genes. These can be derived from other varieties or from non-cultivated materials, including related wild species. Several cycles of backcrossing combined with careful selection allow the original genotype of the cultivated variety to be almost completely recovered even after an initial cross with materials not adapted to cultivation. The relative simplicity of this breeding method is often counterbalanced by the short duration of the resistance as it can be overcome by the pathogen due to mutation of its avirulence genes into virulence genes. This breeding technique therefore involves continuous efforts to find and introduce new resistance genes into cultivated varieties, a process which in breeding is called pyramiding or stacking.

These problems have led to the development of modern biotechnological approaches by plant breeders which have not only attempted to use the resistances available in the genepool to which the crop plant belongs (often helped by Marker Assisted Selection approaches) but also to use genetic transformation aimed at the development of transgenic plants resistant to biotic stresses or herbicide molecules produced by the expression of genes often of microbial origin. Examples are the resistance to lepidoptera pests introduced into corn and resistance to glyphosate introduced in soybean.

The 5 papers published in Frontiers in Plant Science for the Research Topic *Biotechnology Solutions for Crop Production* are relevant to this interesting area. The first four papers report experimental data that take into consideration the use of biotechnological techniques to promote resistance to pathogens (cyst nematode in soybean and quick wilt in black pepper), the attempt to use decomposition leachates of milk vetch for weed suppression, and the use of CRISPR-Cas9 to confer resistance to glyphosate in soybean. The fifth paper concerns an analysis of the impact of Bt cotton in an Indian district and the relationships between sustainable agriculture and GM crops. All five papers were published by Chinese and Indian researchers, partly active at research institutes in the United Kingdom and the USA; this would seem to indicate that this type of approach towards new technologies is particularly important for plant breeders from the two largest Asian Countries, the most populous in the world and characterized by a strong rate of technological development and therefore naturally interested in being able to apply modern technologies in food production.

The paper on cyst nematode (Sultana et al.), conducted at US research institutions, concerned the functional analysis of cyst nematode inducible synthetic promoters in transgenic soybean, to provide insights into the potential applicability of synthetic promoters engineering for conditional expression of transgenes leading to GM crop development for resistance improvement in soybean.

The paper on black pepper (Indu et al.), from India and United Kingdom, concerned the analysis of the consequences of priming in modulating defense against quick wilt in black pepper, a crop of economic interest for India. Glycol Chitosan was used to infiltrate detached leaves of mature black pepper that resulted in significant reduction of disease symptoms in infected leaves. Experiments repeated in black pepper seedlings under controlled growth conditions suggested a priming-associated systemic defense response.

Chinese results to understand the action of decomposition leachates of milk vetch for goosegrass suppression is reported in the third paper (Liu et al.). Milk vetch is a plant widely planted in the temperate zone of China as a green manure to enrich soils that also appears to have potential for goosegrass suppression. The study highlighted three compounds associated with goosegrass suppression potential produced during the decomposition process of milk vetch, a result which can provide a reference for biological weed control, a potentially interesting result in organic farming.

The use of CRISPR-Cas9 to confer resistance to glyphosate in soybean is the target of the fourth paper (Sony et al.), developed at the International Center for Genetic Engineering and Biotechnology, New Delhi, India. This paper describes the development of glyphosate-resistant rice lines by site-specific amino acid substitutions and modification of phosphoenolpyruvate-binding site in the native gene. The authors highlighted that the efficacy of the introduced mutations has contributed to glyphosate resistance, enhanced aromatic amino acids and improved seed yields in rice.

The latest paper, produced by an Indian researcher active in the United Kingdom (Subramanian), is unlike the others and does not report data produced by the author. Instead he analyzed the effect of the introduction of Bt cotton in the Ballari district of India. According to the author, based on his analysis of the data, the yield results show that, in the most recent decade, Bt cotton yields have stagnated, have a null effect on profits, and have produced cotton lines more sensitive to other pest pressures. This paper demonstrates that often single breeding approaches on their own do not produce sustainable improvements in crop production but need to be part of integrated pest management within sustainable farming systems. This paper could push for these improvements, by encouraging pyramiding of resistance sources to a wider range of pests in order to improve production stability in GM crops in the medium to long term.

Overall, the papers published within the Research Topic Biotechnology Solutions for Crop Production are interesting even if, for practical application in the development of improved varieties, they will need to be developed over a suitable number of years. The use of agronomic systems that use the ability of specific plants to suppress weed flora is undoubtedly to be taken into careful consideration, just as the effects that management of GM plants can have in the long term must be carefully monitored. Plant breeders require advanced tools and to carefully study the impacts of these new genetic breeding methods so as to homogenize traditional breeding with the new advanced genetic technologies.

## Author contributions

JS: Writing – original draft, Writing – review & editing. FV: Writing – original draft, Writing – review & editing.

